# Enhanced diagnosis and severity assessment of carpal tunnel syndrome using combined shear wave elastography and cross-sectional area analysis: A prospective case-control study

**DOI:** 10.1371/journal.pone.0320011

**Published:** 2025-03-24

**Authors:** Jaewon Kim, Min-Wook Kim, Jae Min Kim

**Affiliations:** Department of Rehabilitation Medicine, Incheon St. Mary’s Hospital, College of Medicine, The Catholic University of Korea, Seoul, Republic of Korea; King Abdulaziz University, SAUDI ARABIA

## Abstract

Carpal tunnel syndrome (CTS) is a prevalent neuropathy resulting from median nerve compression, typically diagnosed through electrodiagnostic studies. Shear wave elastography (SWE) has emerged as an essential imaging technique, especially in evaluating tissue elasticity, which could enhance the diagnosis and severity assessment of CTS. This study aimed to examine the combined effect of the median nerve’s cross-sectional area (CSA) and elasticity assessed through SWE in diagnosing CTS and evaluating its severity. A total of 50 participants were involved in this prospective study, with 99 wrists analyzed (51 affected by CTS and 48 normal controls). Measurements of both CSA and elasticity were taken at the carpal tunnel inlet. The findings indicated that CSA and elasticity were considerably higher in CTS patients than in the controls. The combined parameter of CSA ×  elasticity outperformed other measures for differentiating between normal and CTS cases (Area Under the Curve 0.91, sensitivity 0.90, specificity 0.83, cutoff 753.7 kPa·mm²). This combined metric also showed potential for distinguishing CTS severity levels, particularly between mild and severe cases. Although CSA and elasticity alone had limitations in severity classification, their combined values illustrated significant distinctions across severity levels. Integrating SWE with CSA notably improves diagnostic accuracy for CTS and shows potential for severity grading. This approach offers a more detailed evaluation of the structural and mechanical changes in the median nerve, potentially enhancing both the diagnosis and management of CTS.

## Introduction

Carpal tunnel syndrome (CTS) impacts approximately 14% of the global population and is among the most common neuropathies resulting from median nerve compression within the carpal tunnel. This condition contributes significantly to medical expenses and socioeconomic challenges [1,2]. Traditional diagnostic approaches consist of nerve conduction studies (NCS) and electromyography (EMG), which evaluate the electrical activity within muscles and nerves. The electrodiagnostic study (EDX), encompassing NCS and EMG, is considered the gold standard for diagnosing CTS and classifying its severity, showing over 85% sensitivity and 95% specificity [3]. Some patients might skip the test due to discomfort, while others may have additional conditions like polyneuropathy or myopathy that complicate diagnosing or assessing the severity of CTS. Moreover, EDX mainly assesses large Aβ fibers but does not provide insights into smaller sensory Aδ or C fibers [4].

As a result, complementary diagnostic methods are necessary, with ultrasound (US) currently being the most common alternative. The US carries multiple benefits, such as being cost-effective and allowing real-time visualization of the median nerve. This technique also aids in identifying anatomical nerve disorders. The median nerve’s cross-sectional area (CSA) at the wrist is the most frequently used measurement [5]. Quantitative CSA measurements sometimes do not align well with patient symptoms or disease severity, and they do not adequately show the acute or chronic course of the condition.

Additionally, US cannot evaluate the mechanical properties of the nerve, such as its stiffness [6]. Furthermore, analyzing nerve echogenicity is qualitative. Given the heterogeneous and anisotropic characteristics of the nerve, echogenicity varies significantly based on factors like the angle of the US transducer and the manipulation by the operator [7,8]. These limitations frequently lead to inconsistencies between US findings and CTS diagnosis or severity classification. Researchers are now investigating the use of shear wave elastography (SWE) on the median nerve to overcome these issues.

Elasticity indicates how a material can revert to its original shape after being deformed by an external force. In the context of nerves, elasticity is quantified in meters per second (m/s) or kilo-Pascals (kPa). SWE is a non-invasive US-based imaging method that assesses tissue stiffness, showing promise in the CTS diagnosis [9–12]. It assesses tissue elasticity by measuring particle displacement following the transmission of an acoustic beam from an US transducer [13,14]. In the SWE, the transducer creates an acoustic radiation force when positioned over the targeted area. This force causes mechanical vibrations to spread laterally through the tissue. The US equipment captures and measures these vibrations, creating a shear wave propagation speed map. This technique offers quantified elasticity data, relying on the principle that shear waves move more quickly through stiffer and denser tissues [15,16]. The results are presented as a 2D image. Unlike strain elastography, which heavily relies on operator input and is sensitive to transducer pressure, SWE offers a more objective and quantitative assessment of tissue elasticity without requiring external pressure. Previous research has thoroughly investigated the CSA of the median nerve, and recent results indicate that elasticity may serve as a valuable parameter [10,12,17,18]. In patients with CTS, pressure from the surrounding bony structures and tendons under the transverse carpal ligament leads to edema, fibrosis, and increased elasticity. However, clinical evidence backing its employ needs strengthening, and only some studies simultaneously assessed CSA and elasticity. Hence, this study explores the roles of US parameters, particularly nerve CSA and elasticity assessed by SWE, in diagnosing and grading CTS severity. Additionally, by employing a multi-beam SWE technique along with the Reliable Measurement Index (RMI) system to gauge measurement reliability, we aim to enhance the accuracy of elasticity readings and improve diagnostic precision.

## Methods

### Ethical considerations

This prospective study at a single center received approval from the Institutional Review Board of Incheon St. Mary’s Hospital (No. OC23DISS0016). The study was conducted by the Declaration of Helsinki. All participants provided written informed consent prior to their enrollment.

### Study participants

This case-control study was conducted between 03 July 2023 and 29 February 2024, focusing on patients aged 20–74 seeking treatment in the Department of Rehabilitation Medicine. The study included individuals with clinically confirmed CTS, diagnosed through EDX tests that showed median nerve abnormalities at the wrist, along with symptoms typical of CTS. The NCS and EMG results classified the wrists according to the American Association of Neuromuscular & Electrodiagnostic Medicine criteria into control and CTS groups, categorized by severity: mild, moderate, and severe [19]. The healthy control group included adults without clinical symptoms in the wrist or hand and who displayed normal results on NCS. We excluded any patient with a history of wrist or finger surgery, injection treatments to the wrist or upper limb over the last 3 months, past trauma, or systemic diseases impacting neuromuscular function (such as diabetes, polyneuropathy, rheumatoid arthritis, and renal disease). Pregnant participants were also excluded.

The sample size calculation was based on the results of a previous clinical study using the following formula [20].


n1=σ12+σ12kz1−α2+z1−β2Δ2,n2=kn1

$$\Delta =\left|\mu_{1}-\mu_{2}\right|,$$

$$\text{k}=\frac{n_{2}}{n_{1}}$$

where $n_{1}$is the size of control group with $\left(\mu_{1},\sigma_{1}\right)$ distribution, $n_{2}$the size of patient group with $\left(\mu_{21},\sigma_{12}\right)$. Assuming a control group mean of 32, variance of 10, patient group mean of 39, a sample size ratio k of 1.7, a type I error (α) of 0.05, and a type II error (β) of 0.1. The required sample sizes were estimated as 34 normal wrists for the control group and 58 symptomatic wrists for the patient group. For each subject, scans of both wrists were performed. For CTS subjects, symptoms could be present in one or both wrists. Based on previous clinical study findings and accounting for an estimated dropout rate of approximately 15%, the final sample sizes were adjusted to 20 for control group (40 wrists) and 30 (60 wrists) for patient group.

### US system

**Fig 1:**
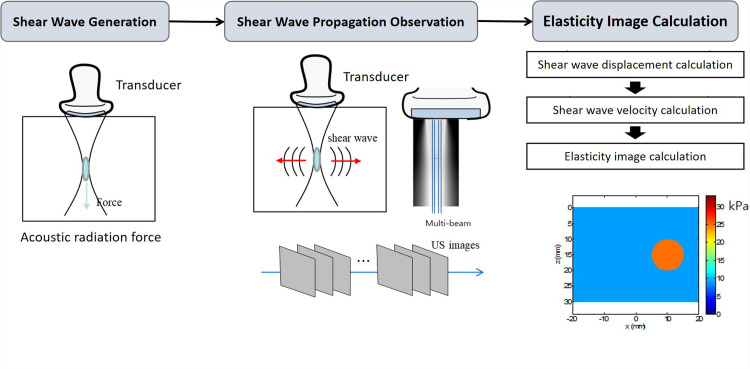
Key Principles of Multibeam Shear Wave Elastography (SWE).

The US examinations were conducted with the Samsung V8 US system (Samsung Medison, Seoul, Korea), which includes a linear array transducer (LA2-14A probe, Samsung Medison Co., Ltd.). The S-Shearwave Imaging™ feature was used for SWE implementation, generating shear waves and monitoring their movement through a multibeam observation method to evaluate tissue elasticity, as illustrated in Fig 1.

The transducer produces shear waves by applying the acoustic radiation force to the targeted area. A multi-beam receiver monitors these shear waves, calculating their displacement and velocity to generate the elasticity image.

**Fig 2:**
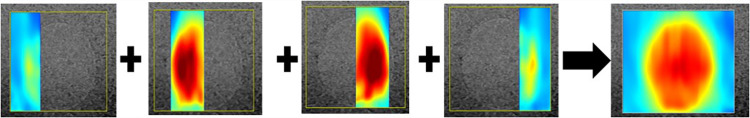
Process of Generating Multibeam-Based Shear Wave Elastography Images.

The multibeam observation technique produces a narrower shear wave imaging area. To address this limitation, Fig 2 demonstrates how several shear wave elasticity images have been stitched together to form a composite shear wave elasticity map. Moreover, the system includes an RMI, which displays a color-coded reliability map of the elasticity measurements. This configuration improves diagnostic accuracy and user convenience.

The region of interest (ROI) is segmented into parts to create several elastography images merged into the final image.

The SWE functions in split-screen mode, featuring the RMI map next to the semitransparent S-Shearwave Imaging™ overlaid on a gray-scale anatomical image. The RMI map uses a color scale to assign reliability values, with green to yellow showing high reliability and red to black indicating low reliability. Tissue elasticity is represented by color, from blue (soft) to red (hard), and users can adjust this in kilopascals (kPa) using Young’s modulus measurements.

Acoustic radiation force impulses created by several focused points cause tissue deformation, which generates shear waves. These waves enable the real-time measurement of tissue elasticity (stiffness).

### US measurement

Three experienced examiners conducted the US examinations in neuromuscular imaging, featuring one with over 8 years of experience and two with more than 20 years of expertise.

**Fig 3:**
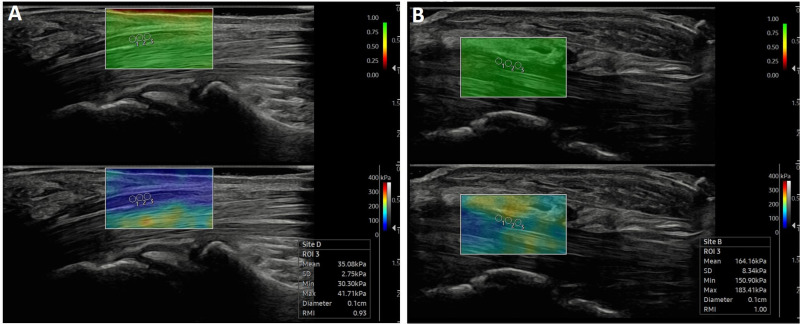
Shear Wave Elastography (SWE) Assessment of the Median Nerve in Normal Individuals (A) and Patients with Severe Carpal Tunnel Syndrome (B). The top images in the US feature the Reliable Measurement Index (RMI), while the bottom images illustrate the elasticity measurements. Measurements were conducted at the carpal tunnel inlet using a 2 mm ROI in three different locations.

Patients were laid supine with their elbows extended, forearms supinated, and wrists in a neutral position to ensure muscle relaxation during the examination. This setup aligns with standard positioning in EDX procedures. A towel was used beneath the wrist to prevent any flexion or extension, providing stabilization. The transducer was applied using free-hand compression, applying minimal pressure to lessen stress on the median nerve in the carpal tunnel and reduce nerve tension. The examination was performed in the following order:

(1) Gray-scale (B-mode) US imaging was utilized to visualize the median nerve and assess its CSA at the carpal tunnel inlet, marked by the proximal edge of the flexor retinaculum (transverse carpal ligament).
(2) SWE was used to evaluate the elasticity of the median nerve. SWE images were captured in the longitudinal planes of the median nerve at the carpal tunnel inlet, with a 90° rotation to achieve the longitudinal view. The region of interest (ROI) was defined using three 2-mm round sub-ROIs, concentrating on the nerve’s central portion while avoiding areas next to the bone or transverse carpal ligament.
(3) Elasticity measurements were recorded from three ROIs at the carpal tunnel inlet, with adjustments made to ensure the clarity of the elasticity images by referencing the RMI (Fig 3).

### Clinical data collection

Patients’ demographic information—age, sex, and body mass index (BMI)—was recorded. Furthermore, clinical data, including pain intensity (Numerical Rating Scale, NRS), symptom severity, and functional status, were collected utilizing the Boston Carpal Tunnel Questionnaire (BCTQ) and symptom duration.

### Statistical analysis

Pearson’s correlation coefficient assessed the relationships among continuous variables such as median nerve CSA, elasticity, NRS scores, and BCTQ scores. Correlation coefficients were interpreted as negligible (0.00–0.10), weak (0.10–0.39), moderate (0.40–0.69), strong (0.70–0.89), and very strong (0.90–1.00) based on established guidelines [21]. Normality was tested using the Shapiro-Wilk test. Effect size measures were calculated using Hedges’ g to evaluate differences in CTS severity (normal, mild, moderate, severe). Independent t-tests were conducted for variables that followed a normal distribution. We assessed the diagnostic performance of median nerve CSA, elasticity, and CSA  ×  Elasticity for CTS—specifically sensitivity, specificity, and optimal cutoff values—using receiver operating characteristic (ROC) curve analysis. The Mann-Whitney U test was used to compare the total CTS group with the normal control group, as normality was not assumed. Bonferroni correction was applied for intergroup comparisons. A Bonferroni-adjusted significance threshold of P <  0.016 was used to control for Type I errors.

## Results

### Study participants

This study included 50 participants: 30 patients and 20 controls. A total of 99 wrists were assessed; however, one wrist was excluded due to a missing NCS measurement. Additionally, during the diagnostic process, some wrists in the patient group were found to be healthy. As a result, the total number of normal wrists was 48, comprising 40 from the control group and 8 from the patient group (5 left wrists and 3 right wrists). The remaining 51 wrists were diagnosed with CTS (24 left wrists and 27 right wrists).

CTS cases were classified by severity as follows: 22 mild, 20 moderate, and 9 severe (Table 1). The overall mean RMI for all cases was 0.91 (SD 0.08), indicating good SWE quality.

**Table 1:** Baseline characteristics of study participants.

Characteristic	Patient Group (n = 30)	Control Group (n = 20)
Age (SD), yr	58.5 (10.1)	54.7 (13.6)
Sex (Male/Female)	13/ 17	7/ 13
Height (SD), cm	161.7 (8.4)	162.5 (9.4)
Weight (SD), kg	69.3 (13.0)	59.5 (10.2)
BMI (SD)	26.4 (4.1)	22.5 (3.3)
Affected Side		
Right	6	
Left	3	
Both	21	
Symptom Duration (SD), mo	33.5 (40.2)	
Pain intensity, NRS (SD)	2.6 (2.2)	
BCTQ-SS (SD)	38.3 (12.6)	
BCTQ-FS (SD)	23.3 (9.5)	

### BMI, body mass index; BCTQ, Boston Carpal Tunnel Questionnaire; SS, symptom severity; FS, Functional Status scale. Normality test and effect size measure

Shapio-Wilk normality test results are shown in Table 2 below where (w,p) values are shown for both control and CTS group on CSA, Elasticity, and CSA  ×  Elasticity. W values are high but p-values are lower than 0.05, and it is normality of the data can not be established.

**Table 2:** Normality test.

Variable	Group	w-value	p-value
CSA	Control	0.894	<0.001
	CTS	0.962	0.102
Elasticity	Control	0.896	<0.001
	CTS	0.917	0.002
CSA × Elasticity	Control	0.758	<0.001
	CTS	0.862	<0.001

Effect size measures are presented in Table 3, where Hedges’ g is used: $g_{1}$represents Hedges’ g, and $g_{2}$is the bias-corrected version of $g_{1}$. The effect sizes were sufficiently large across all three parameters. Additionally, a post-hoc power analysis confirmed that statistical power was adequate, with all power values exceeding 0.98.

**Table 3:** Effect size measure (Hedge’s g).

Parameter	Group define	$g_{1}$	$g_{2}$
CSA	normal vs CTS	1.64	1.61
	≤ mild vs higher	1.44	1.41
	≤ moderate vs severe	1.1	1.08
Elasticity	normal vs CTS	1.09	1.07
	≤ mild vs higher	0.98	0.96
	≤ moderate vs severe	1.32	1.29
CSA × Elasticity	normal vs CTS	1.3	1.27
	≤ mild vs higher	1.24	1.22
	≤ moderate vs severe	1.52	1.5

### CSA analysis

**Fig 4:**
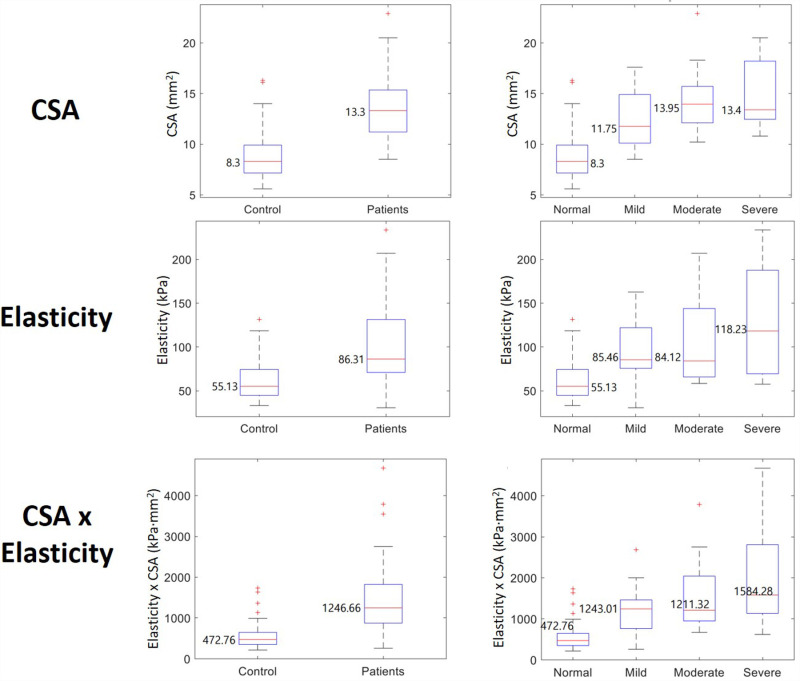
Comparison of Cross-Sectional Area (CSA) and Elasticity in Control and CTS Groups.

Wrists affected by CTS showed a significantly greater CSA than those of normal control wrists (13.6 ±  3.1 mm² compared to 9.0 ±  2.5 mm², g2 = 1.61, t-test p < .001). The normal group had a markedly smaller CSA than all CTS severity groups (p < .001). However, no significant CSA differences were found among the severity levels within the CTS group (p =  0.182 for mild vs. moderate, p =  0.165 for mild vs. severe, p =  0.962 for moderate vs. severe) (Table 4 and Fig 4).

**Table 4:** Comparison of Cross-Sectional Area (CSA) and elasticity across groups.

	Median nerve with carpal tunnel syndrome (CTS)				Normal median nerve (n = 48)	p -value^*^
	Mild degree (n = 22)	Moderate degree (n = 20)	Severe degree (n = 9)	CTS Total (n = 51)		
CSA, mm^2^	12.5 (2.7)	14.3 (3.0)	14.9 (3.4)	13.6 (3.1)	9.0 (2.5)	<.001
Elasticity, kPa^†^	95.5 (32.6)	105.8 (48.0)	133.7 (62.3)	106.0 (47.2)	61.0 (21.8)	<.001
CSA x elasticity, kPa·mm^2^	1223.5 (570.9)	1515.6 (809.0)	2074.5(1332.3)	1488.2 (856.3)	551.6 (337.2)	<.001

*Compared between the total CTS group and the normal control group using the Mann-Whitney U test.

† Elasticity measured in the longitudinal plane.

### Elasticity analysis

The CTS group showed much greater elasticity compared to the normal control group (106.0 ±  47.2 kPa vs. 60.96 ±  21.8 kPa, p < .001). In contrast, the normal group displayed significantly less stiffness than the CTS severity groups (p < .001 for control vs. moderate and severe, p =  0.002 for control vs. mild). Nevertheless, no significant differences in elasticity were observed among the various severity levels within the CTS group (p =  0.805 for mild vs. moderate, p =  0.05 for mild vs. severe, p =  0.241 for moderate vs. severe) (Table 4 and Fig 4).

### Combined CSA and elasticity analysis

The CSA ×  elasticity measurement revealed a notable difference between the CTS and control groups, with median values of 1246.7 kPa·mm² for the patient group compared to 472.8 kPa·mm² for the control group (g2 = 1.27, p <  0.001). This combined parameter indicated that the severe group had significantly higher values than the mild group (p =  0.006). However, the moderate group did not exhibit statistically significant differences from either the mild or severe groups (p =  0.451 for mild vs. moderate, p =  0.134 for moderate vs. severe) (Table 4 and Fig 4).

### Receiver operating characteristic (ROC) analysis

**Fig 5:**
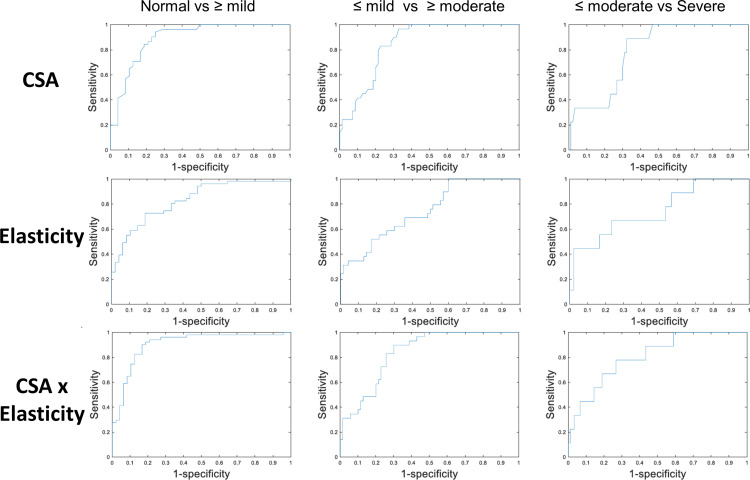
Receiver Operating Characteristic (ROC) Curve for US Measurement of Cross-Sectional Area (CSA), Elasticity, and the CSA x Elasticity.

The ROC analysis for CSA showed an Area Under the Curve (AUC) of 0.89, with a sensitivity of 0.94 and specificity of 0.75, using a cutoff of 9.9 mm² to distinguish between normal subjects and those with mild or more severe CTS. Regarding elasticity, the AUC stood at 0.83, achieving a sensitivity of 0.73 and specificity of 0.81 with a cutoff value of 75.7 kPa. When combined, the CSA ×  elasticity parameter surpassed individual measures, yielding an AUC of 0.91 for differentiating normal subjects from those with CTS, with sensitivity at 0.90 and specificity at 0.83 at a cutoff of 753.7 kPa·mm². Additionally, this combined parameter presented an AUC of 0.84 for differentiating mild from moderate or greater CTS and an AUC of 0.81 for distinguishing between moderate or milder and severe cases. The ROC analysis for predicting severe cases indicated a cutoff value of 1226.0 kPa·mm², with a sensitivity of 0.78, specificity of 0.73, and an accuracy of 0.77 (Table 5, Fig 5).

**Table 5:** Receiver Operating Characteristic (ROC) and cut-off values of the US parameters.

	Cut-off value	Sensitivity	Specificity	Accuracy	PPV	NPV	AUC	p -value
Normal vs CTS								
CSA, mm^2^	9.9	0.94	0.75	0.84	0.78	0.93	0.89	<.001
Elasticity, kPa	75.68	0.73	0.81	0.77	0.78	0.76	0.83	<.001
CSA x elasticity, kPa·mm^2^	753.71	0.90	0.83	0.87	0.84	0.90	0.91	<.001
≤ mild vs ≥ moderate CTS								
CSA, mm^2^	10.8	0.97	0.67	0.88	0.88	0.89	0.85	<.001
Elasticity, kPa	76.45	0.69	0.64	0.68	0.82	0.46	0.75	<.001
CSA x elasticity, kPa·mm^2^	863.26	0.90	0.70	0.84	0.88	0.74	0.84	<.001
≤ moderate vs severe CTS								
CSA, mm^2^	12.3	0.89	0.68	0.87	0.97	0.38	0.79	.002
Elasticity, kPa	95.04	0.67	0.77	0.68	0.97	0.19	0.75	<.001
CSA x elasticity, kPa·mm^2^	1225.97	0.78	0.73	0.77	0.97	0.25	0.81	<.001

### Correlation analysis

A weak positive linear relationship between elasticity and CSA was identified, indicated by an R² value of 0.26 (p < .001). Additionally, both CSA and elasticity exhibited weak correlations with clinical features, such as the NRS, as well as the Boston Carpal Tunnel Questionnaire Symptom Severity (BCTQ-SS) and Functional Status (BCTQ-FS) scales. Specifically, the correlations for CSA were R² =  0.19, 0.24, and 0.16, all statistically significant (p < .001). For elasticity, the correlations were R² =  0.05 (p =  0.024), 0.15 (p < .001), and 0.10 (p < .001).

## Discussion

This study found that integrating SWE elasticity measurements with median nerve CSA achieved the highest diagnostic accuracy, with an AUC of 0.91 for differentiating between normal and CTS. Additionally, to distinguish between moderate or milder and severe CTS, combining elasticity and CSA resulted in an AUC of 0.81, indicating a potential synergistic advantage in considering both structural and mechanical characteristics of the median nerve when evaluating advanced stages of CTS.

Our findings indicate that CSA and elasticity were notably greater in CTS patients than controls, reinforcing earlier studies that documented increased CSA and elasticity in CTS groups vs. healthy individuals. This research identified the diagnostic cutoff for CSA as 9.9 mm², exhibiting a sensitivity of 0.94 and a specificity of 0.75, corresponding with the previously documented cutoff range of 9–12 mm² [5,22,23]. The diagnostic cutoff value for elasticity in CTS is 75.7 kPa, with a sensitivity of 0.73 and a specificity of 0.81. These results align with previous studies’ findings [20,24,25].

Although CSA is commonly employed in the US to diagnose CTS, its effectiveness in evaluating the severity of CTS is still debatable [26]. While greater CSA has been noted in patients with CTS compared to controls, the evidence indicating a consistent correlation between CSA and CTS severity is limited and inconsistent. Some research has identified elevated CSA in more severe instances [27], while others found no significant correlation between CSA and CTS severity [20,28,29]. Consistent with earlier research, our findings indicate that CSA does not significantly vary with the severity of CTS.

Additionally, we observed no noteworthy differences in elasticity across the different levels of CTS severity. Like CSA, past studies have produced mixed results about the link between elasticity and CTS severity, with some reporting no significant differences and others showing conflicting outcomes [30]. For example, some studies observed severe cases exhibited much greater elasticity than mild or moderate cases, with values of 101 kPa compared to 55.1 kPa [20,24,25]. Conversely, some observed a notable rise in elasticity among the moderate and severe groups compared to the mild cases [31]. Certain studies revealed a notably greater elasticity in severe cases than in mild ones [10]. A systematic review indicated that elasticity might help distinguish between various levels of CTS severity [32].

Studies show that median nerve elasticity cutoff values for diagnosing CTS vary significantly, falling between 28.2 kPa and 79 kPa [33–36]. This variation stems from multiple factors, such as differences in selecting the ROI, probe pressure during the examination, and closeness to the bone, which can result in elevated elasticity values due to the “bone proximity hardening artifact.” Furthermore, variations among US systems can cause notable differences in elasticity measurements, with studies indicating discrepancies of up to 26% between different devices [37–39]. Additionally, variations may depend on factors such as tissue characteristics (e.g., size and shape), probe application technique, ROI depth, methodology, study population, and disease severity [40,41]. Validating the reproducibility of SWE values still needs more work. However, by assessing elasticity (or stiffness), SWE reflects tensile loading during mechanical stretching, which makes it a helpful tool for examining peripheral nerve pathology from a mechanical viewpoint [42].

This study examined the diagnostic benefits of multiplying CSA and elasticity values. While there may be differing opinions on this method, our goal was to evaluate its straightforward utility for clinicians during US examinations. Some previous studies indicate that combining CSA with SWE enhances sensitivity and specificity or significantly aids in distinguishing between severity levels [43,44].. Our results demonstrate that the CSA ×  elasticity measure outperforms individual parameters, achieving a cutoff of 753.7 kPa·mm² (AUC =  0.91, sensitivity =  0.90, specificity =  0.83) in differentiating normal and CTS cases. Although CSA and elasticity alone showed limitations in grading severity, their combination revealed significant differences, particularly in severe cases (p <  0.01). The established cutoff for severe CTS was 1226.0 kPa·mm², with a sensitivity of 0.78 and specificity of 0.73, highlighting its clinical relevance. However, the sensitivity and specificity metrics may have been influenced by the spectrum and severity of cases included. Higher sensitivity, as observed for normal vs. CTS, likely reflects the inclusion of cases with distinct severity levels, facilitating clearer discrimination. Conversely, lower specificity in ≤  mild vs. ≥  moderate CTS may be attributed to overlapping features between mild and moderate cases. These findings underscore the need for broader case distributions in future studies to more comprehensively evaluate diagnostic performance in diverse clinical settings.

Histological analysis of the median nerve in CTS patients revealed that areas affected by repetitive compression experienced significant demyelination and remyelination, along with considerable loss of large, myelinated axons. Furthermore, regions exhibiting nerve swelling showed heightened perineurial and endoneurial fibrosis [45]. Swelling progresses from proximal to distal areas affected by compression, accompanied by fibrosis resulting from chronic inflammation. In the areas where compression is localized, there is a thinning of the myelin, significant thickening and contraction of the internal epineurium, and increased thickening of the endoneurial microvessels, epineurium, and perineurium. Large myelinated fibers exhibit severe myelin thinning and an elevated axon-to-myelin and axon-to-fiber ratio [46]. The histological alterations in the median nerve during CTS enhance nerve elasticity. In particular, initial changes in fibers occur within the peripheral fascicles, advancing to the central fascicles as CTS progresses [46]. The histological findings explain the increasing CSA x elasticity values observed with greater severity in this study.

We enhanced accuracy and reproducibility by employing the RMI function of the US device. RMI quantifies the intensity of shear waves generated in the tissue and assesses how uniformly these waves propagate, indicating the reliability of elastography measurements. By incorporating RMI during the SWE scans, we achieved highly reliable elastographic images and accurately measured the elasticity values. We also generated higher-quality and more precise elastographic images using a US device with a multibeam observation method focusing US energy into a narrow zone.

This study has various limitations. First, interrater and interrater reliability assessments were not conducted. Nevertheless, a highly skilled physician carried out the US examinations, and the RMI values from the US system were referenced, indicating a minimal margin of error. Second, measurements were solely taken at the carpal tunnel inlet, neglecting individual differences in CSA and elasticity. Third, the anatomical features of the nerve may have led to anisotropy. While attempts were made to reduce mechanical anisotropy by maintaining the transducer’s perpendicular position during the median nerve elasticity measurements, this limitation underscores the inherent difficulties in assessing peripheral nerves. Additionally, it is important to recognize that anisotropy may indicate structural disorganization within the peripheral nerve [7]. Fourth, the relatively small sample size, coupled with the case-control design with a high prevalence of CTS cases, may have led to an overestimation of positive and negative predictive values (PPV and NPV), thereby limiting the generalizability of these findings. Increasing the sample size would enhance the robustness and generalizability of the results. Nonetheless, despite this limitation, the observed trends remain clear and meaningful, supporting the validity of our findings. Future studies with larger cohorts are recommended to further confirm these results and strengthen their clinical implications.

Currently, the EDX is still the gold standard for diagnosing CTS and for deciding between surgical and nonsurgical treatment. However, in situations where EDX cannot be conducted or when underlying neuromyopathies require further evaluation, growing research on US parameters, including SWE, may improve its usefulness in classifying severity. This could help in developing suitable treatment approaches, particularly in differentiating between surgical and nonsurgical options.

## Conclusion

SWE provides essential insights into the median nerve’s mechanical properties, and when paired with CSA, it greatly enhances the diagnostic accuracy for CTS. The combined CSA ×  elasticity measure exceeded the effectiveness of individual metrics in differentiating normal cases from CTS and exhibited promise in distinguishing various degrees of CTS severity. Integrating SWE with CSA allows a more thorough evaluation of the median nerve’s structural and mechanical changes, potentially improving diagnosis and severity classification.

## Supporting information:

S1 Data Raw data for statistical analysis.
(XLSX)
